# Nurse-Training Schools and Registration

**Published:** 1888-08-04

**Authors:** 


### The Hospital

A Weekly Institutional Journal of

**Science, Medicine, Nursing, and Philanthropy.**

*For Hospitals, Asylums, Medical Practitioners, Students, Nurses, Families, Parishes, Congregations, and the Charitable Public.*

Vol. IV. No. 97.] [August 4, 1888.

### Nurse-Training Schools and Registration.

Mr. Henry Bonham-Carter, honorary secretary to the Nightingale Fund, discussed last week in our columns the question of the desirability of establishing a single general register for trained nurses. The conclusion arrived at by the writer was that on the whole such a register is undesirable and might be injurious both to the public and to nurses; whilst even if its desirability in the abstract can be maintained, the time for its establishment has not yet come. A general register, such as is contemplated, to be of any value must be carried out by charter and under State authority. But it is obvious that some public justification is required before the machinery of the State can properly be invoked. Thoughtless and inexperienced persons may see no reason why the State should not be moved in the interests of individuals or of particular classes. But those who have any rational appreciation of the proper functions of the central authority know how undesirable, nay, how seldom justifiable it is for the State to depart from established custom in order to legislate for the advantage, or supposed advantage, of individuals or of classes. We repeat that the interference of the State can only be properly invoked and justified by proved public necessity or advantage.

Is there then a proved public necessity for the State registration of trained nurses? This question must not be shirked. Mr. Bonham-Carter seems to be of opinion that not only is there no proved public necessity for a registration scheme, but that, on the contrary, there might result from its establishment a whole series of practical difficulties of a markedly injurious character.

The medical register is not unnaturally looked to as offering an example of the benefits of general registration. Concerning this Mr. Bonham-Carter says: "How far a legal register is essential or advantageous in the case of doctors I will not stop to inquire. Some of the highest authorities in the profession have expressed more than doubts about it." It is easy to emphasise this statement by repeating the well-known fact that so far from the bulk of the medical profession seeing any advantage to themselves in the register, many of them regard it not only as an inconvenience and a nuisance, but as an engine of extortion and oppression. The registration office extracts a large fee from the young doctor on his first qualification, and, so far as he can discover, does not confer one single practical benefit upon him during the whole of his career. If there be any advantage, it is not to the practitioner, though he alone has the privilege of paying the bill.

We are, however, discussing now the question, not of advantage or disadvantage to the nurse, but to the public. Such a registration scheme as is proposed would probably result, Mr. Bonham-Carter thinks, in the elevation of the more educated but less practical nurse, and the consequent depression of the less cultured but more practically efficient one. That would be a grievous injury all round—to the nurse first of all, but much more to the public. It cannot be too steadfastly affirmed that the business of a nurse is to nurse. She must be able and willing to do that, whatever else she leaves undone. In every calling culture and good manners have their advantages, and may add to the value of special qualifications; but they can never take the place of them. The business of a doctor is not to be a classical scholar, or an antiquarian, or even a scientist, but a medical practitioner. Health and disease, diagnosis and treatment, pills and powders, nursing and feeding,—these he must have at his fingers' ends. If, in addition he be a scholar, a gentleman and a man of the world, so much the better. But when it comes to the real management of disease, it is the man who understands his business who is placed in charge, even if it be Hutton the bonesetter.

It is precisely so with nurses. A nurse must nurse whether she drops her h's or not, whether she is pretty or plain, whether her caps and aprons look charming or ugly, whether she has been bred in the drawing-room or in the scullery. Mr. Bonham-Carter thinks, and vast numbers of people who know take the same view, that a general register would put a premium on mere book knowledge, whilst practical efficiency, because of the impossibility of testing it by examinations, would become of less and less account. The public would suffer because they would be inefficiently nursed, and nursing as an art would deteriorate in exact proportion to its diminished practicality. That is no fancy picture. If any man can speak with weight and authority, it is Mr. Bonham-Carter. If the appeal be made to the whole medical profession, what will the answer be? Physicians and surgeons will affirm with one voice—"We want, first of all, nurses who can nurse, whether they be ladies or kitchenmaids."

Mr. Bonham-Carter thinks, and we are entirely at one with him, that those who train nurses, who observe them at actual work for a term of years, are alone able to report on and adjudicate their value. If they are to be registered at all, let it not be under the exclusive jurisdiction of a central body, which will merely read written answers to printed questions, and see each candidate for ten minutes or a quarter of an hour, or even less. What can such a body really know about them? As is pointed out in Mr. Bonham-

Carter's paper, the moral factor is one of the most important of all as regards the character and fitness of a nurse. What cognisance could a central registration authority take of the morals of a nurse at the East or West End of London, at Manchester, at Edinburgh, at Penzance, or at John o'Groats? Every hospital worth the name, and every private nursing institution of any standing at all, knows, values, and registers accordingly every nurse under its charge. This is a registration based on knowledge, on years of experience. A certificate emanating from such a source has a definite value. All practical people know this. It is of the very essence of plain every-day sense that it should be so.

We have not by any means exhausted Mr. Bonham-Carter's objections to a central registration scheme, nor would it be possible to do so in the space at our disposal. But it is important to point out that whilst to put a nurse on the register would be comparatively easy, to remove her from it would, in many cases of urgent necessity, be quite impossible. After registration a woman might, by gradual deterioration of character, become absolutely unfit for the peculiar duties of sick nursing, or even for associating with decent people; and yet she might avoid such gross indecencies as would justify her removal from the register. It is well known that there are numbers of medical men who are not worthy to enter a virtuous person's house, and yet any man attempting their removal from the register would, in most instances, entirely fail of his purpose, and bring upon himself an infinity of expense and trouble. The power of discipline in a general register such as is contemplated would be practically *nil*. On the other hand the disciplinary power of hospitals and well-conducted nursing institutions is, in effect, all that can be wished. Moreover this disciplinary authority is found to be perfectly compatible with the largest freedom any well-conducted woman can desire.

Such are a few of the important issues raised by Mr. Bonham-Carter's eminently practical paper. It seems certain that the more sober and far-seeing among matrons, nurses, and hospital managers have, by their own methods of reasoning, arrived at similar conclusions. No doubt there is a fine sound about the words "a learned profession," and equally, no doubt, a certain number of those who are captivated by phrases and sounding titles would like not merely to be members of the "nursing profession," but to have authority, emolument, and status therein. But the great world has something else to think about, and Government has something else to do than to concern itself with organisations which a year ago did not exist, and a year hence may have vanished into nothingness. In the past hospitals have steadily improved the training and skill of their nurses, and nurses have as steadily made use of their opportunities. The public has looked on with approval, and has expressed its satisfaction in a thousand ways. The hospitals will continue to improve their training, and the nurses will rise higher and higher in public esteem and confidence. This they will do by their own increasing merit and the growing value of their services to the world. If in the course of years it should be found that the general average of culture and practical skill is so high as to make a central register possible and desirable, such a register will doubtless be established. In the meantime it seems not only best, but imperative that the hospitals and nursing institutions themselves shall each keep its own register and be responsible for the character and well-being of its own nurses.

